# Effects of a Combined Intradialytic Exercise Training Program and Music on Cardiac Autonomic Nervous System Activity in Hemodialysis Patients

**DOI:** 10.3390/life12081276

**Published:** 2022-08-20

**Authors:** Maria Mitsiou, Eleftherios Dimitros, Stefanos Roumeliotis, Vassilios Liakopoulos, Evangelia Kouidi, Asterios Deligiannis

**Affiliations:** 1 Laboratory of Sports Medicine, Aristotle University of Thessaloniki, 57001 Thessaloniki, Greece; 2 Division of Nephrology and Hypertension, 1st Department of Medicine, AHEPA Hospital, Medical School, Aristotle University of Thessaloniki, 54636 Thessaloniki, Greece

**Keywords:** intradialytic exercise, hemodialysis, music, heart rate variability, functional capacity

## Abstract

This study aimed to examine the effect of an intradialytic exercise program in combination with music on heart rate variability (HRV) indices and functional capacity in patients on maintenance hemodialysis (HD). Methods: Forty HD patients were randomized to four training groups for six months: the combined music and exercise group (Group A), the exercise group (Group B), the music group (Group C), and the control group (Group D). At baseline and after 6 months, all participants underwent both short- (for 30 min) and long- (for 24 h) term measurements of HRV and functional capacity assessment with a 6 min walking test (6MWT). Patients of groups A and C listened to preferred music. Results: Long-term HRV analysis showed that standard deviation of all normal-to-normal RR intervals (SDNN) and the square root of the mean squared differences of successive RR intervals (rMSSD) were significantly higher at the end of the study in groups A (by 13.2% and 47.3%), B (by 15.1% and 50%), and C (by 9.0% and 30.1%), compared to group D (*p* < 0.05). Values of rMSSD and percentage of RR intervals differing by more than 50 ms from the preceding RR interval (pNN50) were elevated in groups A (by 35.6% and 142.9%), B (by 36.1% and 75%), and C (by 15.2% and 28.6%), compared to baseline measurements (*p* < 0.05). Also, pNN50 was increased in group A compared to groups B (by 21.4%), C (by 88.9%), and D (by 142.9%) (*p* < 0.05). Similar results were noted by short-term HRV analysis. Functional capacity was improved at the end of the 6-month study in groups A (by 20.3% and 25.7%) and B (by 15.8% and 21.1%) compared to groups C and D (*p* < 0.05). Conclusions: Intradialytic exercise combined with music-listening can improve the functional capacity and cardiac autonomic nervous system activity in hemodialysis patients.

## 1. Introduction

Chronic kidney disease (CKD) is considered a leading global public priority, with increasing incidence and prevalence over the last two decades. Although it is difficult to calculate the precise prevalence of CKD, it is estimated that about 11–15% of the population worldwide is affected by CKD [1]. Through its impact on end-stage kidney disease (ESKD) and cardiovascular (CV) disease (CVD), CKD is directly associated with increased mortality and morbidity worldwide [2], and by 2040, it is forecasted to become the fifth leading cause of death [3].

Cardiac autonomic nervous system (CANS) dysfunction results from increased sympathetic and decreased parasympathetic activities [4] and is associated with severe arrhythmias and sudden cardiac death in patients with heavy CV burden, such as those with coronary heart disease [5]. Likewise, in ESKD, patients undergoing maintenance hemodialysis (HD) or CANS dysfunction predisposes for both CVD and sudden cardiac death, and therefore, timely identification is crucial [6].

Heart rate variability (HRV), derived from digitalized electrocardiographic signals, is an indirect, noninvasive measurement for assessing cardiac sympathetic and parasympathetic activity [7]. It is reported that HRV is severely de-arranged in CKD patients [8] and becomes even more pronounced in HD. In these patients, HRV is thought to cause sympathetic hyperactivity and vagal withdrawal, possibly leading to left ventricular hypertrophy [9] and development of complex arrhythmias [8,10]. Various pathophysiological mechanisms underlying this effect have been proposed, including reduced arterial baroreflex function, activation of the renin–angiotensin–aldosterone system (RAAS), activation of renal afferents fibers, CV remodeling, altered nitric oxide bioavailability, mental stress [10], insulin resistance, increased endothelin production, salt retention, and activation of atrial natriuretic peptide [4,7].

Various strategies have been proposed to ameliorate the deleterious effects of autonomic dysfunction on HD patients, including the use of RAAS inhibitors, the use of b-receptor blockers, and physical exercise [7]. It has been reported that intradialytic exercise might improve indices of HRV, thus resulting in reduction of CV disease [11,12]. Moreover, aerobic exercise in HD patients has been reported to improve functional capacity [13,14,15] and quality of life [16,17].

On the other hand, listening to music is known to induce feelings of pleasure, elevate mood, and reduce stress, possibly through the stimulation of various brain regions, including the dopamine system. Since the beneficial effects of music to human mental health are undisputed, it has been used in intervention therapeutic programs [18]. Besides the mental–psychological benefits, it has been shown that pleasant music may also enhance parasympathetic activity in resting conditions [19] and improves post-exercise parasympathetic reactivation, leading to faster recovery and reduced cardiac stress after exercise [20]. Furthermore, preferential music combined with exercise significantly improves autonomic balance by inducing changes in HRV [21]. In CKD patients, several studies have reported that music might exert a significant beneficial effect on pain and anxiety [22], reduce heart rate and systolic and diastolic blood pressure [23], and improve pain and nausea during HD sessions [24]. However, the impact of music combined with exercise intervention on autonomic regulation in ESKD patients undergoing maintenance HD remains unclear. Hence, this study aims to evaluate long-term effects of music along with exercise on HRV indices in HD patients.

## 2. Materials and Methods

### 2.1. Study Participants

**Figure 1:**
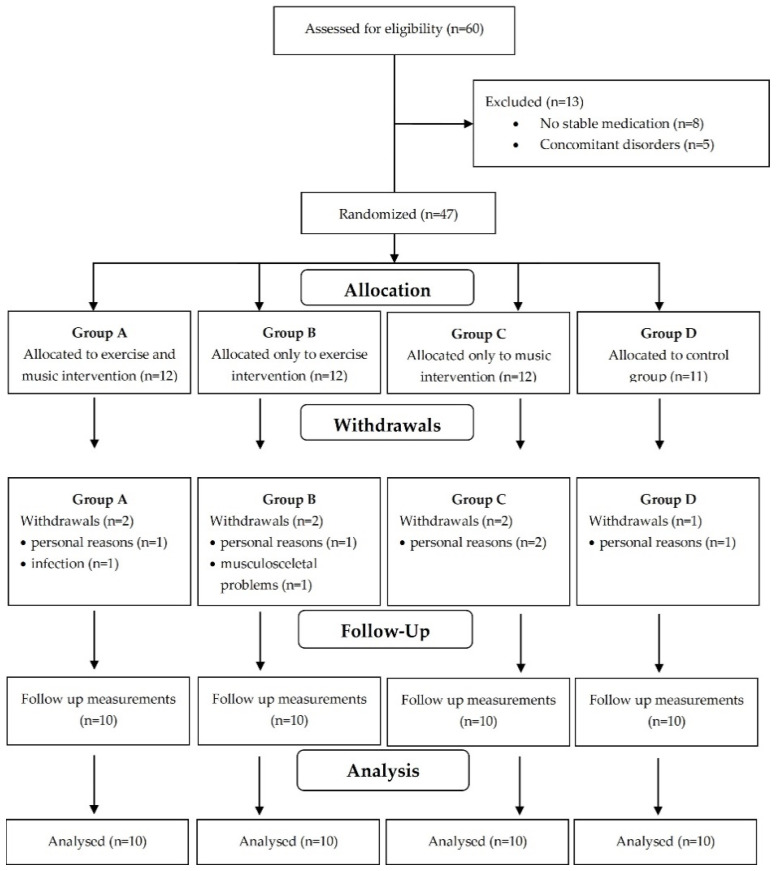
Flowchart of study design.

For this randomized controlled trial, all eligible adult patients who underwent HD in the Renal Unit of AHEPA Hospital were asked to participate in the study. No transplant recipients were included in the study, but all the participants were candidates for receiving a kidney transplant. Sixty ESKD patients undergoing maintenance HD three times a week for 4 h were initially assessed for enrollment. Inclusion criteria for the study were age of over 18 years, ESKD, and receiving HD for at least 3 months. The exclusion criteria of this study included concomitant severe cardiac diseases, musculoskeletal problems, severe anemia, current infection, medication that affects the ANS (such as cholinomimetics/cholinesterase antagonists (e.g., edrophonium), anticholinergics (e.g., ipratropium and tiotropium), adrenoreceptor agonists/sympathomimetics (e.g., clonidine) or adrenoreceptor antagonists sympatholytic (β-blockers or α-agonists)), stimulant medications such as amphetamines or methylphenidates, and life expectancy of less than 6 months. After the initial evaluation, eight patients were excluded from the study due to unstable medication and five patients because they fulfilled one or more exclusion criteria. The study design and flowchart are presented in Figure 1. Forty-seven patients were enrolled and randomized by a simple random allocation method (drawing lots) to four training groups for six months: the combined music and exercise group (Group A), the exercise group (Group B), the music group (Group C) and the control group (Group D). Allocation secrecy was kept by numbered, sealed, opaque envelopes. During the follow-up period, seven patients withdrew from the study; thus, 40 patients (10 in each group) completed the study. All participants were informed about the purpose, the procedures, and the possible benefits of the study and provided their written informed consent. The Ethics Committee of the Department of Physical Education and Sport Science of Aristotle University of Thessaloniki approved the study protocol with registration number 83/2021, which was conducted according to the Declaration of Helsinki.

### 2.2. Study Design and Intervention

At enrollment, blood samples were drawn from all participants to determine hematologic and biochemical parameters. Additionally, all patients underwent interview for their medical history, physical examination, and 12-lead electrocardiogram (ECG). At the beginning and the end of the 6-month study, all patients were assessed for functional capacity (FC) with a 6 min walking test (6MWT), long-term measurement of HRV with 24-h ambulatory ECG monitoring, and acute short-term measurement HRV during exercise using a heart rate monitor. In addition, patients were advised to abstain from smoking, coffee, and alcohol for at least 12 h before measurements. All tests at baseline and the end of the 6-month study were performed by a single well-trained researcher who was blinded to the identity of patients to avoid bias.

The 6-month supervised exercise training program for group A and group B was conducted three times a week during the first two hours of their HD sessions in a semi-recumbent position using a static bicycle (Motomed Letto 713/W 1498, Tampa, FL, USA), adjusted to the bed. Patients who participated in the exercise rehabilitation program cycled for 30 to 60 min with gradually increased intensity corresponding to a 12–13 (medium to hard) rating of perceived exertion on the 6–20 Borg scale. The first five minutes was the warm-up, followed by 20 to 50 min of cycling and 5 min of cool-down, including stretching exercises of lower limbs. During the training sessions, heart rate (HR) and blood pressure (BP) were monitored and recorded. The exercise intensity and duration were individually prescribed, progressively increased, and readjusted according to each patient’s ability and physical condition.

Music as an intervention was used both in groups A and C during the 6-month study duration. Patients listened to preferred music through a portable player (mp3 player) using in-ear headphones for 30 to 60 min during the first two hours of their HD sessions. During this time period, the acute short measurement of HRV was also conducted. Initial and final measurements of acute short measurement of HRV were performed using the same music tracks. Group D followed their usual lifestyle routine without participating in any exercise program or physical activities that could affect the results. The researchers conducting the data processing and analysis were blinded to the intervention assignments.

### 2.3. Functional Capacity

Since the 6MWT is a reliable tool reflecting functional performance in HD patients [25], we used it to estimate the functional capacity and muscle endurance of the lower limbs of the patients, according to guidelines of the American Thoracic Society [26]. Participants were required to walk for 6 min as fast as possible to their own pace on a 30 m marked flat track. The total distance covered after the completion of the test was recorded.

### 2.4. 24 h Measurement of HRV

Indices of HRV were analyzed by 24 h ambulatory ECG monitoring of patients using a 3-channel ECG digital Holter recorder (Vision 5L, Burdick, Bothell, WA, USA). Data were stored on a memory card and then analyzed using Burdick Vision Premier Software (Burdick, Bothell, WA, USA) to obtain the heart rate variability as the standard deviation (SD) of all the regular R–R intervals (SDNN). All ectopic beats and artifacts were removed both automatically and manually by a blinded investigator. Spectral analysis was based on the fast Fourier transform (FFT), providing the total (0.01–1.00 Hz), low (0.04–0.15 Hz), and high (from 0.15 to 0.40 Hz) frequency power. Patients were instructed to follow their usual daily activities and avoid any form of exercise, alcohol, and caffeine consumption that could affect HR during recording.

For the estimation of HRV, both time and frequency analyses were obtained, according to Task Force recommendations [27]. The time-domain analysis included several HRV indices, including standard deviation of all normal-to-normal RR intervals (SDNN), reflecting the total sympathovagal balance, square root of the mean squared differences of successive RR intervals (rMSSD), and the proportion derived by dividing the number of interval differences of successive RR intervals more significant than 50 milliseconds by the total number of RR intervals (pNN50).

From frequency domain analysis, the HRV indices evaluated were low-frequency power (LF), expressed in normal units (representing both sympathetic and parasympathetic activity); the high-frequency power (HF), expressed in normal units (representing mainly parasympathetic activity); the LF-to-HF ratio (LF/HF), also expressed in normal units (to estimate autonomic balance); and the meager frequency power (VLF), indicating sympathovagal balance, as described before [27,28].

### 2.5. Acute Short-Term Measurement of HRV

HR values were recorded by Polar S810i^TM^ (Polar Electro Oy, Kempele, Finland) for 30 min at the beginning of the intradialytic exercise program. This device can monitor the beat-to-beat HR for HRV analysis. Specifically, the instrument was used to capture RR intervals through a transmitter attached to an elastic band fitted around the thorax. Then, the signals were continuously transmitted and stored in a receiver via an electromagnetic field for further analysis and evaluation of HRV indices [29]. Then, data were analyzed by Polar Precision Performance Software (Polar Electro, Kemple, Finland) to correct values and exclude artifacts. Finally, HRV indices were derived using Kubios HRV analysis software (Kuopio, Finland). Time-domain and frequency domain indices of HRV included those described on ambulatory ECG monitoring.

Geometric methods of HRV included the triangle interpolation of the histogram of intervals NN (TINN). The TINN consists of measuring the base of a triangle determined by the method of least squares [30]. This index expresses the overall variability of RR intervals [27] and is affected more by low than high frequencies.

Non-linear measures for determining HRV indices included the Poincaré plot. In addition, SD1 and SD2 were analyzed by calculating the standard deviations of distances from RR intervals [30]. SD1 is reported to be sensitive to parasympathetic nervous system activity, while SD2 reflects overall HRV and is sensitive to both sympathetic and parasympathetic tones [31].

### 2.6. Statistical Analysis

Continuous variables were expressed as mean ± SD and were all normally distributed. Shapiro–Wilk test was used to examine the normality distribution. Assumption of the homogeneity of variances was analyzed using Levene’s test. Two-way ANOVA with repeated measures of variance was used to detect changes within and between the groups at baseline. At the end of the study, time and group served as independent variables. Where a significant F was observed, a *t*-test for paired samples was used for detecting differences within groups. One-way ANOVA using Tukey post hoc test was performed to investigate significant differences between groups. A two-tailed *p*-value of *p* < 0.05 was considered statistically significant. For the statistical analyses, we used the Statistical Package for Social Sciences version 25.0 software for Windows (IBM Corp., Armonk, NY, USA).

## 3. Results

### 3.1. Participants’ Characteristics

Initially, 60 HD patients were assessed for eligibility and 47 patients were included in the study. During the follow up period, seven patients withdrew from the study; thus, 40 patients, 20 males and 20 females (10 to each group), managed to complete the study without any complications or reported side-effects. The mean age of the patients included in the study was 50 ± 12.7 years, and patients were randomized to the four groups: Group A, 6 males and 4 females aged 48.5 ± 15.4 years; Group B, 4 males and 6 females aged 50.6 ± 10.8 years; Group C, 5 males and 5 females aged 50.5 ± 11.5 years; and control Group D, 5 males and 5 females aged 50.2 ± 14 years old. The demographic and clinical characteristics of participants are shown in Table 1.

### 3.2. Ambulatory 24-h Holter monitoring

The results of HRV indices obtained from 24 h Holter recording at baseline and follow-up are shown in Table 2. The analysis showed that at the end of the study, HR was decreased in all intervention (A, B, and C) groups by 7.5%, 5.7%, and 5,6% respectively (*p* < 0.05), while no significant difference was found for group D (*p* > 0.05). At the end of the study, regarding time-domain analysis, SDNN was significantly increased in groups A, B, and C compared to group D by 13.2%, 15.1%, and 9.0%, respectively (F_3, 36_ = 11.671, *p* < 0.05). Moreover, it was improved significantly in groups A, B, and C compared to baseline values by 15.4%, 18.4%, and 6.4% (*p* < 0.05). The follow-up findings of rMSSD showed an increase in groups A, B, and C compared to group D by 47.3%, 50%, and 30.1%, respectively (F_3, 36_ = 12.395, *p* < 0.05). Furthermore, rMSSD was significantly higher in all intervention groups A, B, and C compared to baseline by 35.6%, 36.1%, 15.2%, respectively (F_3, 36_ *p* < 0.05) and decreased in group D by 11.8% (*p* < 0.05). At the end of the study, pNN50 was increased in group A compared to groups B, C, and D by 21.4%, 88.9%, and 142.9% (F_3, 36_ = 45.752, *p* < 0.05). It was also increased in group B compared to groups C and D by 55.6% and 100.0% (*p* < 0.05). Moreover, it was higher in groups A, B, and C than baseline values by 142.9%, 75%, and 28.6%, respectively (*p* < 0.05). At the start of the study, HFnu was significantly higher in group B than groups C and D by 42.9% and 40.8% (F_3, 36_ = 4.236, *p* < 0.05). At the end of the study, it was decreased in group B compared to initial measurements by 24% (*p* < 0.05). At baseline, LFnu was higher in groups A and B than group D by 10.4% and 11.5% (F_3, 36_ = 7.608, *p* < 0.05). Moreover, during follow-up measurements, it significantly decreased in groups A and B by 9.7% and 8%, respectively (*p* < 0.05).

### 3.3. Acute HRV Indices

The findings from the acute short-term measurement of HRV indices are presented in Table 2. SDNN was higher in group A compared to group B at baseline by 14.7% (F_3, 36_ = 4.606, *p* < 0.05) and in groups A, B, and C compared to group D at the end of the study by 45.3%, 23.7%, and 21.2%, respectively, (*p* < 0.05). It was also increased in group A compared to groups B and C by 17.4% and 19.9% (*p* < 0.05). Moreover, after the follow-up period, SDNN increased in groups A, B, and C by 31.0%, 28.1%, and 12.2%, respectively (*p* < 0.05). We also found that pNN50 was higher at the end of the study in group A compared to groups C and D by 20.8% and 31,8% (F_3, 36_ = 11.297, *p* < 0.05) and in group B compared to group D by 18.2% (*p* < 0.05). Additionally, it was higher in groups A, B, and C after the intervention compared to initial measurements by 26.1%, 18.2%, and 4.3% (*p* < 0.05) and lower in group D by 4.3% (*p* < 0.05). At baseline, rMSSD was lower in group A compared to groups B, C and D by 17.5%, 24.3%, and 30.5% (F_3, 36_ = 37.260, *p* < 0.05). After the follow-up period, rMSSD was increased in groups A, B, and C by 35.7%, 19.8%, and 8.2%, respectively (*p* < 0.05), whereas, after the 6-month period, VLF was higher only in group B compared to D by 28.7% (F_3, 36_ = 3.141, *p* < 0.05) and higher in groups A and B compared to baseline values by 21.2% and 10.6% (*p* < 0.05).

### 3.4. Functional Capacity

**Figure 2:**
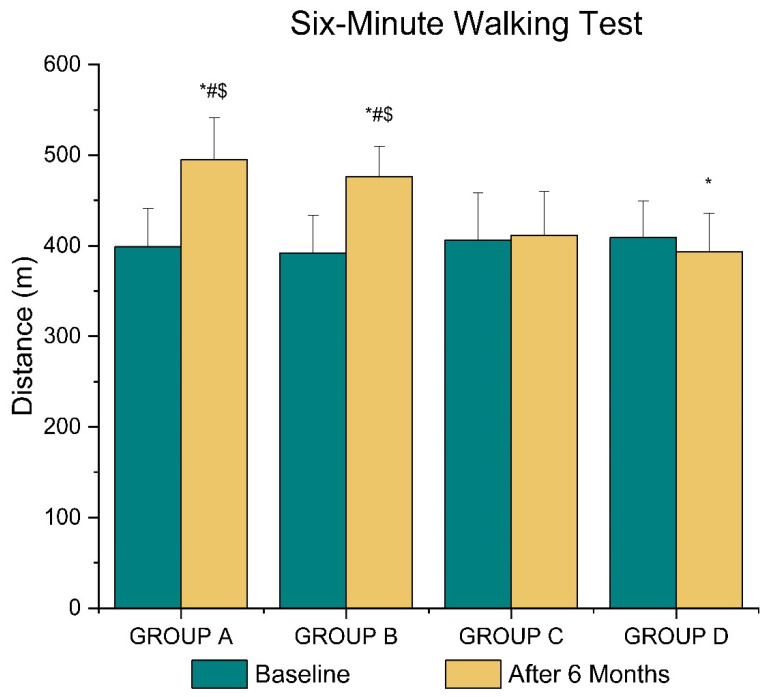
Walking distance values for study groups at baseline and after the 6-month intervention programs. * *p* < 0.05 vs. baseline, ^#^ *p* < 0.05 vs. Group C, ^$^ *p* < 0.05 vs. Group D. Group A: exercise and music intervention, Group B: exercise intervention, Group C: music intervention, Group D: control group.

Figure 2 shows the differences in walking distance among study groups at baseline and after the 6-month intervention programs. The 6MWT at the end of the protocol was higher in group A compared to groups C and D by 20.3% and 25.7% (F_3, 36_ = 13.095, *p* < 0.05) and in group B compared to groups C and D by 15.8% and 21.1%, respectively (*p* < 0.05). Moreover, it was increased in groups A and B and decreased in group D during follow-up compared to baseline values by 24.0%, 21.5%, and 3.8%, respectively (*p* < 0.05).

## 4. Discussion

This study shows the beneficial effects of a six-month intradialytic exercise training program combined with music on CANS activity and functional capacity of HD patients. Since CANS dysfunction is associated with mortality and CVD, these findings are of clinical importance [32].

The increased sympathetic and the reduced parasympathetic activity in HD patients are attributed to cardiac autonomic dysfunction, anemia, reduced aerobic capacity, and uremic autonomic dysfunction [33,34,35]. Exercise training, especially aerobic programs, causes alterations in cardiac autonomic activity by enhancing resting vagal tone and decreasing sympathetic tone [36]. In this study, baseline values representing vagal activity were reduced while indices of sympathetic activity were increased. After a 6-month intradialytic exercise training program, the time and frequency domain HRV indices were enhanced in normal units, indicating improved vagal activity leading to sympathovagal balance. Similarly, other studies have also reported significant changes in HRV following an exercise training program. It has been reported that SDNN, RMSSD, HF, and LF components were augmented after six months of exercise training in both HD patients and kidney transplant recipients [11,37,38]. There is evidence that physical exercise training also improves HRV indices in patients with diabetes [39,40], heart failure [36], or cancer [41]. However, in disagreement with our findings, other studies have reported no changes in autonomic cardiac control after exercise intervention in patients undergoing HD, indicating that ANS is not altered during the course of a 4 h HD session program [42] or after a short 12-week exercise training period [43,44]. It should be noted that in these studies, exercise was examined solely and not the combination of exercise with music.

It is noted that HRnu was decreased in the exercise training group. It is possible that the level of exercise intensity may have different results on cardiac autonomic function. For example, high-intensity endurance training was more effective in improving parasympathetic activity compared to low-intensity endurance training in healthy individuals [45]. Low HRV is associated with an elevated risk of all-cause mortality. Additionally, there are no standard guidelines regarding the extent to which HRV needs to improve to reduce CV risk [45]. Moreover, exercise characteristics like training duration, frequency, intensity [44,46,47], and total duration of the exercise program, as well as physical fitness, seem to play a significant role on HRV [38,45].

The data regarding short HRV indices response during intradialytic exercise are very limited. In a recent study, Kaltsatou et al. [42] found that pupillometry indices, related to parasympathetic activity, were correlated with HRV parameters indicating vagal activity such as pNN50, rMSSD, and HF after 15 min from the end of the exercise, confirming the beneficial effects of exercise on cardiac function. In our study, acute measurements of SDNN, pNN50, and rMSSD were all elevated after the 6-month follow-up in all three intervention groups, reflecting the benefits of both exercise and music. It is well-known that SDNN represents a general estimation of autonomic balance modulated both by sympathetic and parasympathetic branches, while rMSSD and pNN50 are mainly affected by vagal tone [48]. It has been demonstrated that there is continued sympathoexcitation beyond 50–60% of maximal oxygen consumption without further decrease in parasympathetic activity and that in healthy populations, parasympathetic effects persist during high-intensity exercise [49]. Moreover, it has been suggested that reduced parasympathetic tone during exercise may be an essential factor of increased risk for sudden cardiac death. In contrast, an augmented vagal tone may have a protective role in the occurrence of sudden death [50].

The exact mechanisms leading to these changes in HRV indices are still unclear. One possible explanation might be the impact of exercise training on decreased catecholamine levels at rest and during submaximal exercise [36]. Another potential mechanism underlying this might be the suppression of angiotensin II, leading to vagal tone inhibition [51]. Nitric oxide bioavailability may also increase parasympathetic and decrease sympathetic activity [52], especially in CKD and ESKD patients [53]. Other possible explanations include the improvement of endothelial function via exercise [54] and intrinsic sinus node adaptations [46]. Short-term exercise might also cause autonomic adaptations decreasing sympathetic and increasing parasympathetic activity, leading to bradycardia, while long-term aerobic exercise might cause atrial and ventricular dilatation [55] and thus could induce intrinsic electrophysiological adaptations and increase parasympathetic activity [46].

The findings of the present study have shown that music can enhance parasympathetic tone in maintenance HD patients. Similarly, Archana et al. [21] investigated the effect of music on HRV in 30 healthy young adults and found that preferred music has a calming impact on exercise, causing changes in HRV. The effect of music on HRV parameters has been thoroughly examined in a recent systematic review [56]. The authors included 29 studies in their analysis, examining the effect of musical interventions on HRV in a heterogenous population, including healthy subjects, patients with neuropsychiatric diseases, patients undergoing surgery, dental procedures, or cardiac catheterization. They concluded that music may act as a stimulus that could enhance parasympathetic activity and HRV. Moreover, it has been shown that music, besides increasing parasympathetic tone and minimizing the rise in heart rate, might also reduce fatigue during low exercise intensity [57]. In disagreement with these, other studies have shown that music has no impact on vagal tone during exercise [20]. A study investigating the effect of music on anxiety and vital parameters of CKD patients found significant improvement in anxiety scores, systolic and diastolic BP, HR, and respiratory rate. The authors reported that music could be used in clinical practice as a possible low-cost intervention to improve mood, reduce anxiety, and improve BP [23].

The 6MWT has been repeatedly used to assess the FC of ESKD patients. It has been estimated that the average 6 min walking distance for dialysis patients is about 458 m [58]. Vogiatzaki et al. [59] found that a 6-month intradialytic aerobic exercise program increased the physical performance of HD patients as measured by 6MWT, leading to improvement of dialysis adequacy. In their study, Torres et al. [60] observed an increase in walking distance of CKD patients after a 3-month exercise training intervention. Similarly, other studies implementing exercise rehabilitation programs have also found improvements in the functional capacity of CKD patients [61,62]. This finding is significant, as CKD patients can improve their autonomy, personal independence, and social life [63].

Our study has certain limitations that should be addressed, including the small sample size, the different types of music that were used as intervention according to the preference of each participant, and the fact that we did not evaluate or document information regarding dry weight or frequent 24 h ambulatory blood-pressure monitoring. Finally, our cohort does not reflect the typical HD population, which is primarily of old age and with limited capacity for physical activity.

## 5. Conclusions

In conclusion, implementing a 6-month exercise rehabilitation program combined with music hearing is able to cause significant improvements in cardiac autonomic nervous system function by increasing HRV indices, especially those of time domain analysis, reflecting parasympathetic activity. Furthermore, it improves functional capacity as measured by 6MWT. Thus, aerobic exercise combined with music during hemodialysis could be recommended as a standard clinical practice in patients with CKD.

## Figures and Tables

**Table 1:** Demographic and clinical characteristics (mean ± SD) of participants included in the study.

	Group A (n = 10)		Group B (n = 10)		Group C (n = 10)		Group D (n = 10)	
	Baseline	Follow-Up	Baseline	Follow-Up	Baseline	Follow-Up	Baseline	Follow-Up
Sex (male/female)	6/4	-	4/6	-	5/5	-	5/5	-
Age (years)	48.5 ± 15.4	-	50.6 ± 10.8	-	50.5 ± 11.5	-	50.2 ± 14.4	-
HD duration (years)	8.9 ± 2.8	-	8.8 ± 2.7	-	8.7 ± 2.7	-	8.2 ± 2.5	-
CKD etiology								
Glomerulonephritis	3 (30%)	-	2 (20%)	-	3 (30%)	-	3 (30%)	-
Polycystic kidney disease	2 (20%)	-	2 (20%)	-	2 (20%)	-	3 (30%)	-
Nephrosclerosis	1 (10%)	-	2 (20%)	-	2 (20%)	-	1 (10%)	-
Vesicoureteral reflux	2 (20%)	-	2 (20%)	-	1 (10%)	-	1 (10%)	-
Renal ectopia	1 (10%)	-	0 (0%)	-	1 (10%)	-	0 (0%)	-
Interstitial nephritis	0 (0%)	-	1 (10%)	-	0 (0%)	-	1 (10%)	-
Unknown etiology	1 (10%)	-	1 (10%)	-	1 (10%)	-	1 (10%)	-
Comorbidities								
Cardiovascular disease	3 (30%)	-	2 (20%)	-	3 (30%)	-	2 (20%)	-
Hypertension	7 (70%)	-	8 (80%)	-	7 (70%)	-	8 (80%)	-
Hb (gr/dL)	11.7 ± 2.3	11.1 ± 0.1	11.1 ± 7.8	11.6 ± 1.9	11.8 ± 1.4	12.0 ± 0.9	11.6 ± 3.1	11.7 ± 1.8
Fe (μg%)	88.9 ± 28.3	89.2 ± 28.3	90.5 ± 23.0	90.2 ± 25.7	89.7 ± 24.1	89.0 ± 29.7	90.5 ± 13.2	91.0 ± 11.2
WBC (/mm^3^)	5698 ± 1049	5670 ± 1730	5700 ± 1111	5428 ± 1140	5688 ± 1092	5513 ± 1420	5702 ± 1348	5700 ± 1150
Glucose (mg%)	98.2 ± 13.9	97.8 ± 14.7	98.0 ± 12.8	94.7 ± 16.3	98.2 ± 14.2	98.9 ± 15.3	97.3 ± 12.7	97.2 ± 13.3
Urea (mg%)	138.1 ± 17.8	138.3 ± 20.8	141.3 ± 20.3	140.0 ± 30.0	139.3 ± 20.8	139.1 ± 14.1	140.3 ± 21.4	141.3 ± 27.0
Uric acid (mg%)	5.8 ± 1.9	6.4 ± 1.2	5.8 ± 1.2	5.8 ± 1.0	5.8 ± 1.2	5.8 ± 1.1	5.8 ± 1.3	5.9 ± 1.3
Cr (mg%)	9.9 ± 2.0	9.9 ± 2.2	10.1 ± 1.0	10.2 ± 2.0	9.9 ± 1.0	9.9 ± 1.1	10.0 ± 1.8	10.1 ± 1.9
K (mEq/L)	5.6 ± 1.0	5.5 ± 0.8	5.6 ± 0.9	5.7 ± 1.1	5.6 ± 2.1	5.6 ± 1.9	5.5 ± 1.8	5.6 ± 0.1
Na (mEq/L)	135.3 ± 4.3	136.1 ± 2.1	138.2 ± 1.8	138.2 ± 5.0	136.4 ± 4.3	137.3 ± 3.0	137.5 ± 2.4	138.6 ± 1.2
Ca (mg%)	9.1 ± 0.9	9.1 ± 0.5	9.0 ± 4.2	8.9 ± 0.9	9.1 ± 0.7	9.1 ± 1.3	9.0 ± 4.7	9.0 ± 1.1
P (mg%)	6.1 ± 0.6	6.1 ± 1.9	6.0 ± 2.5	6.0 ± 1.3	6.1 ± 1.6	6.0 ± 1.3	6.0 ± 4.4	6.0 ± 1.3

SD: standard deviation, Group A: exercise and music intervention, Group B: exercise intervention, Group C: music intervention, Group D: control group. Hb: hemoglobin, Fe: iron, WBC: white blood cells, Cr: creatinine, K: potassium, Na: sodium, Ca: calcium, P: phosphorus.

**Table 2:** HRV results (mean ± SD) of 24 h ambulatory recording and short-term measurements during exercise.

		Group A		Group B		Group C		Group D	
		Baseline	Follow-Up	Baseline	Follow-Up	Baseline	Follow-Up	Baseline	Follow-Up
HR	Ambulatory	79.5 ± 8.8	73.5 ± 10.9 *	80.3 ± 5.1	75.7 ± 6.0 *	81.6 ± 6.4	77.3 ± 9.4 *	74.6 ± 7.1	73.8 ± 7.4
	Resting	79.6 ± 10.0	73.7 ± 12.8 *	85.6 ± 10.8	80.7 ± 12.9 *	87.7 ± 10.9	84.0 ± 12.0 *	82.2 ± 5.3	81.9 ± 4.1
SDNN	Ambulatory	82.4 ± 2.2	95.1 ± 3.1 *^,+^	81.7 ± 10.9	96.7 ± 7.9 *^,+^	86.1 ± 3.0	91.6 ± 4.9 *^,+^	84.9 ± 2.1	84.0 ± 3.6
	Acute	65.4 ± 7.0 ^#^	85.7 ± 9.0 *^,#,$,+^	57.0 ± 4.6	73.0 ± 4.1 *^,+^	63.7 ± 6.8	71.5 ± 7.4 *^,+^	58.3 ± 5.2	59.0 ± 4.6
rMSSD	Ambulatory	20.2 ± 2.6	27.4 ± 3.7 *^,+^	20.5 ± 2.2	27.9 ± 5.6 *^,+^	21.0 ± 3.5	24.2 ± 2.8 *^,+^	21.1 ± 2.4	18.6 ± 2.3 *
	Acute	36.7 ± 4.4 ^#,$,+^	49.8 ± 5.2 *	44.5 ± 3.4 ^+^	53.3 ± 3.0 *	48.5 ± 2.6	52.5 ± 2.3 *	52.8 ± 3.5	50.1 ± 4.2
pNN50	Ambulatory	0.7 ± 0.1	1.7 ± 0.3 *^,#,$,+^	0.8 ± 0.2	1.4 ± 0.2 *^,$,+^	0.7 ± 0.1	0.9 ± 0.2 *	0.7 ± 0.1	0.7 ± 0.1
	Acute	2.3 ± 0.2	2.9 ± 0.3 *^,$,+^	2.2 ± 0.3	2.6 ± 0.3 *^,+^	2.3 ± 0.2	2.4 ± 0.1 *	2.3 ± 0.2	2.2 ± 0.2 *
LFnu	Ambulatory	76.6 ± 4.1 ^+^	69.2 ± 3.7 *	77.4 ± 3.2	71.2 ± 2.8 *	73.0 ± 3.2	73.5 ± 3.4	69.4 ± 5.7	70.3 ± 5.1
	Acute	39.9 ± 7.9	35.7 ± 7.5 *	40.4 ± 7.0	38.6 ± 7.2 *	40.9 ± 4.5	42.1 ± 5.0	33.9 ± 4.3	36.4 ± 4.3 *
HFnu	Ambulatory	37.2 ± 11.1	31.7 ± 10.9	40.0 ± 8.4 ^$,+^	30.4 ± 12.6 *	28.0 ± 9.7	25.2 ± 10.4	28.4 ± 8.2	27.1 ± 7.9
	Acute	62.9 ± 9.3	61.0 ± 11.0	60.8 ± 8.7	60.7 ± 6.9	54.3 ± 7.6	55.0 ± 8.0	54.2 ± 6.1	55.8 ± 6.7 *
LFnu/HFn	Ambulatory	2.2 ± 0.7	2.5 ± 1.0	2.0 ± 0.4	2.7 ± 1.0	2.9 ± 1.0	3.3 ± 1.1	2.7 ± 0.9	2.8 ± 0.9
	Acute	0.7 ± 0.2	0.6 ± 0.2	0.7 ± 0.2	0.7 ± 0.3	0.8 ± 0.2	0.8 ± 0.2	0.6 ± 0.1	0.7 ± 0.1
VLF	Ambulatory	228.5 ± 93.3	189.6 ± 54.5	233.1 ± 50.3	196.3 ± 29.5 *	174.7 ± 51.4	174.6 ± 54.4	192.0 ± 28.3	186.6 ± 29.6
	Acute	22.6 ± 4.7	27.4 ± 5.2 *	29.2 ± 6.4	32.3 ± 5.9 ^+^	25.1 ± 4.2	26.4 ± 4.9	24.7 ± 7.3	25.1 ± 6.3
TINN	Acute	362.8 ± 163.3	418.0 ± 128.2	450.0 ± 103.7	509.1 ± 128.8 *	456.7 ± 123.0	468.0 ± 118.8 *	463.7 ± 111.4	434.6 ± 89.1
SD1	Acute	48.6 ± 5.6	53.5 ± 5.2 *	54.3 ± 9.5	63.1 ± 8.4 *^,+^	56.3 ± 10.5	54.3 ± 11.1	51.6 ± 7.1	50.7 ± 6.5
SD2	Acute	57.3 ± 4.3	60.3 ± 5.6	54.7 ± 5.6	60.6 ± 6.4 *	60.3 ± 10.4	57.8 ± 8.6	53.9 ± 5.6	53.4 ± 5.9

SD: standard deviation, Group A: exercise and music intervention, Group B: exercise intervention, Group C: music intervention, Group D: control group, HR: heart rate, SDNN: standard deviation of all normal-to-normal RR intervals, rMSSD: square root of the mean squared differences of successive RR intervals, pNN50: the proportion derived by dividing the number of interval differences of next RR intervals more significant than 50 milliseconds by the total number of RR intervals, LFnu: low-frequency power in normal units, HFnu: high-frequency power in normal units, LFnu/HFnu: ratio of low-frequency power to high-frequency power in normal units, VLF: meager frequency power, TINN: triangle interpolation of the histogram of intervals NN, SD1: standard deviations of distances from RR intervals 1, SD2: standard deviations of distances from RR intervals 2. * *p* < 0.05 vs. baseline, ^#^ *p* < 0.05 vs. Group B, ^$^ *p* < 0.05 vs. Group C, ^+^ *p* < 0.05 vs. Group D.

## Data Availability

The data presented in this study are available on request from the corresponding author. The data are not publicly available due to the privacy of included athletes.
